# [^18^F] Clofarabine for PET Imaging of Hepatocellular Carcinoma

**DOI:** 10.3390/cancers11111748

**Published:** 2019-11-07

**Authors:** Olga Sergeeva, Vladimir Kepe, Yifan Zhang, Galen A. Miller-Atkins, Jonathan D. Keynon, Renuka Iyer, Sandra Sexton, Amad Awadallah, Wei Xin, Yogen Saunthararajah, E. Ricky Chan, Zhenghong Lee

**Affiliations:** 1 Radiology, Case Western Reserve University, Cleveland, OH 44106, USA; oxs57@case.edu (O.S.); yxz137@case.edu (Y.Z.); 2 Nuclear Medicine, Cleveland Clinic, Cleveland, OH 44195, USA; KEPEV@ccf.org; 3 Institute for Computational Biology, Cleveland, OH 44106, USA; gmillera92@gmail.com (G.A.M.-A.); erc6@case.edu (E.R.C.); 4 Biology, Case Western Reserve University, Cleveland, OH 44106, USA; jdk39@case.edu; 5 Medical Oncology, Rowell Park Comprehensive Cancer Center, Buffalo, NY 14263, USA; renuka.iyer@roswellpark.org (R.I.); sandra.sexton@roswellpark.org (S.S.); 6 Pathology, University Hospitals Cleveland Medical Center, Cleveland, OH 44106, USA; aawada@yahoo.com (A.A.); wxx10@case.edu (W.X.); 7 Hematology & Oncology, Cleveland Clinic, Cleveland, OH 44195, USA; saunthy@ccf.org

**Keywords:** hepatocellular carcinoma, PET imaging, tumor proliferation

## Abstract

Clinical diagnosis of hepatocellular carcinoma (HCC) relies heavily on radiological imaging. However, information pertaining to liver cancer treatment such as the proliferation status is lacking. Imaging tumor proliferation can be valuable in patient management. This study investigated ^18^F-labeled clofarabine ([^18^F]CFA) targeting deoxycytidine kinase (dCK) for PET imaging of dCK-dependent proliferation in HCC. Since clinical PET scans showed a high liver background uptake of [^18^F]CFA, the aim of this study was to reduce this liver background uptake. A clinically relevant animal model of spontaneously developed HCC in the woodchucks was used for imaging experiments. Several modifiers were tested and compared with the baseline PET scan: Forodesine, probenecid, and cold clofarabine, all applied before the hot [^18^F]CFA injection to evaluate the reduction in liver background uptake. Application of forodesine before hot [^18^F]CFA injection did not reduce the background uptake. Instead, it increased the background by 11.6–36.3%. Application of probenecid also increased the liver background uptake by 16.6–32.1%. Cold CFA application did reduce the liver background uptake of [^18^F]CFA, comparing to the baseline scan. Combining cold CFA with [^18^F]CFA for PET imaging of liver cancers is a promising strategy, worthy of further clinical evaluation.

## 1. Introduction

Hepatocellular carcinoma (HCC) is the third leading cause of cancer-related death worldwide, and is the only cancer whose incidence is increasing in the United States [1]. Clinical diagnosis of HCC relies heavily on radiological imaging with contrast enhanced CT or MRI for detecting typical vascular patterns. However, these findings are often supplemented by biopsies for histopathology evaluation and confirmation, especially for patients without cirrhosis, where most guidelines recommend biopsy. A significant limitation of these standard imaging modalities is the lack of direct and biological information pertaining to the treatment of liver cancer, such as tumor proliferation. The proliferative status of a tumor can be probed by PET imaging for treatment monitoring, provided a suitable PET radiotracer is identified, particularly for early response before measured reduction in tumor size.

2-chloro-9-(2’-deoxy-2’-[^18^F]fluoro-β-d-arabinofuranosyl)adenine ([^18^F]clofarafine, or [^18^F]CFA), a radiolabeled nucleoside analog, was investigated in this study for PET imaging of cellular proliferation in HCC. The cold (non-radiolabeled) clofarabine (CFA) was approved by the Food and Drug Administration (FDA) in 2004 for the treatment of pediatric refractory acute lymphoblastic leukemia (ALL) [2], and it is a substrate of deoxycytidine kinase (dCK), which is a target of several other anti-cancer pro-drugs such as gemcitabine and decitabine. The higher expression of dCK was found in some HCCs, in contrast to the surrounding liver parenchyma, as confirmed by our bioinformatics analyses, which makes it a potentially viable imaging marker for tracking dCK-dependent proliferation. Previously, 1-(2’-deoxy-2’-[^18^F]fluoro-β-D-arabinofuranosyl)cytosine (D-[^18^F]FAC or FAC) was developed for imaging the same dCK in proliferating lymphoid tissues. However, the long synthesis time of FAC posed a logistic challenge for its clinical applications. [^18^F]CFA, with its straightforward synthesis, offers an alternative for imaging dCK-dependent proliferation [3]. The initial clinical biodistribution study showed a high liver background uptake with [^18^F]CFA [4]. The aim of this study was thus to reduce liver background uptake of [^18^F]CFA for PET imaging of liver cancers.

**Figure 1:**
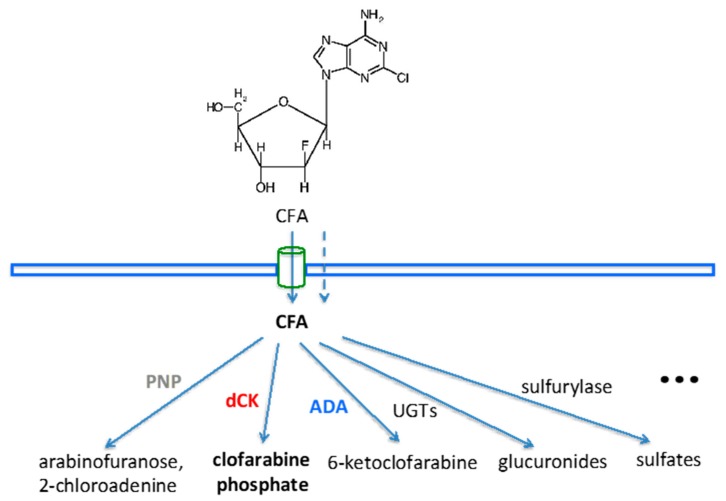
Cellular metabolism and catabolism of clofarabine (CFA). After entering the cell by passive diffusion and/or transport, CFA can be cleaved by purine nucleoside phosphorylase (PNP), although CFA is resistant to PNP cleavage due to the fluorine on the arabinose; or phosphorylated by deoxycytidine kinase (dCK), the preferred metabolic route; or deaminated by adenine deaminase (ADA), even though CFA is resistant to this, due to the chloride on the purine; or glucuronidated into CFA-glucuronides (either CFA-3G or CFA-5G) by uridine 5’-diphospho-glucuronosyltransferase (UDP-GTs or UGTs), abundant in the liver; or metabolized through other routes. All of these would potentially contribute to the high liver background uptake of [^18^F]CFA through the accumulation and retention of radiolabeled CFA metabolites or catabolites.

CFA is transported into the cells by equilibrative nucleoside transporters (ENT1 and ENT2) and concentrative nucleoside transporters (CNT2 and CNT3), plus passive diffusion since it is slightly lipophilic [2]. Retention of cellular CFA depends on its metabolism, shown in Figure 1 [5].

After the uptake into the cells, CFA is converted by dCK to the 5’-monophosphate metabolite, and then by mono- and di-phosphokinases to the active 5’-triphosphate form (CFA-TP). The structure of CFA consists of deoxyadenosine with substitution of hydrogen by chlorine at the 2-position of the adenine ring which causes electronic changes that make the amino group resistant to deamination by adenine deaminase (ADA). Substitution of a fluorine atom at the arabinosyl configuration at the 2’-position of the carbohydrate decreases the susceptibility of CFA to phosphorolytic cleavage by purine nucleoside phosphorylase (PNP), which resides mainly in the cytoplasm of endothelial and Kupffer cells in the liver [6]. In addition, hepatic abundances of uridine 5’-diphospho-glucuronosyltransferase (UDP-GTs or UGTs) would produce two possible CFA glucuronides (CFA-Gs), CFA-5G or -3G. All of the radio-metabolites would possibly contribute to the liver background uptake seen in PET imaging with [^18^F]CFA. We therefore tested the inhibition of PNP and UGTs to exam if there is any reduction in the liver background uptake of [^18^F]CFA using an animal model with clinical relevance.

The eastern woodchuck (*Marmota monax*) can develop HCC after chronic viral hepatitis infection when it harbors a DNA virus—the woodchuck hepatitis virus (WHV), a member of the family Hepadnaviridae, of which human hepatitis B virus (HBV) is the prototype. Like HBV, WHV infects woodchuck to cause acute and chronic hepatitis, which leads to spontaneous development of HCC within 2–4 years of life, with no human tissues or agents involved. The woodchuck HCC is thus considered clinically relevant, with similar pathology and natural history to human HCC [7,8,9]. 

We first performed microarray analysis using the woodchuck tissues to confirm the higher dCK expression in woodchuck HCC than in the surrounding liver parenchyma, as well as higher base main levels of PNP and UGTs in the liver tissues, similar to the analysis of human RNA-seq data from The Cancer Genome Atlas (TCGA). Even though CFA is resistant to hydrolysis by PNP, the high level of PNP expression in the liver might produce some ^18^F-labeled arabinofranose, which would go further down the ribose metabolic pathways in the liver for retention of hepatic radioactivity [10], and contribute to liver background signal. We therefore applied forodesine (immucillin H, or BCX-1777), which is a transition-state analog inhibitor of PNP [11], to test whether its application right before [^18^F]CFA injection would reduce the liver background uptake by decreasing the amount of possible PNP-cleaved radio-metabolite, [^18^F]arabinofuranose in the liver. Forodesine was clinically investigated for the treatment of patients with T-cell acute lymphoblastic leukemia (T-ALL) and for treatment of B-cell acute lymphocytic leukemia (B-ALL) and has been tested in humans with a satisfactory safety profile [12]. 

We also applied probenecid right before [^18^F]CFA injection, intended to reduce the level of CFA glucuronidation for lowering the liver background signal. Probenecid is FDA-approved for the treatment of gout. It inhibits renal excretion of some drugs, thereby increasing their plasma concentration and prolonging their effects [13]. It also inhibits hepatic glucuronidation in a partial non-competitive fashion as exemplified by the inhibition of AZT glucuronidation [14]. In addition, we applied a clinically equivalent dose of cold CFA right before the injection of “hot” [^18^F]CFA. We wanted to test the saturation of hepatic enzymes responsible for CFA metabolism with cold CFA to reduce possible radio-metabolites of [^18^F]CFA in the liver. A higher level of dCK in HCC would lead to a higher metabolic rate than the surrounding hepatic tissues, and would be less affected by the saturation with CFA to display a better contrast uptake of [^18^F]CFA for PET imaging of dCK-dependent proliferation in HCC.

## 2. Results

### 2.1. Bioinformatics

**Figure 2:**
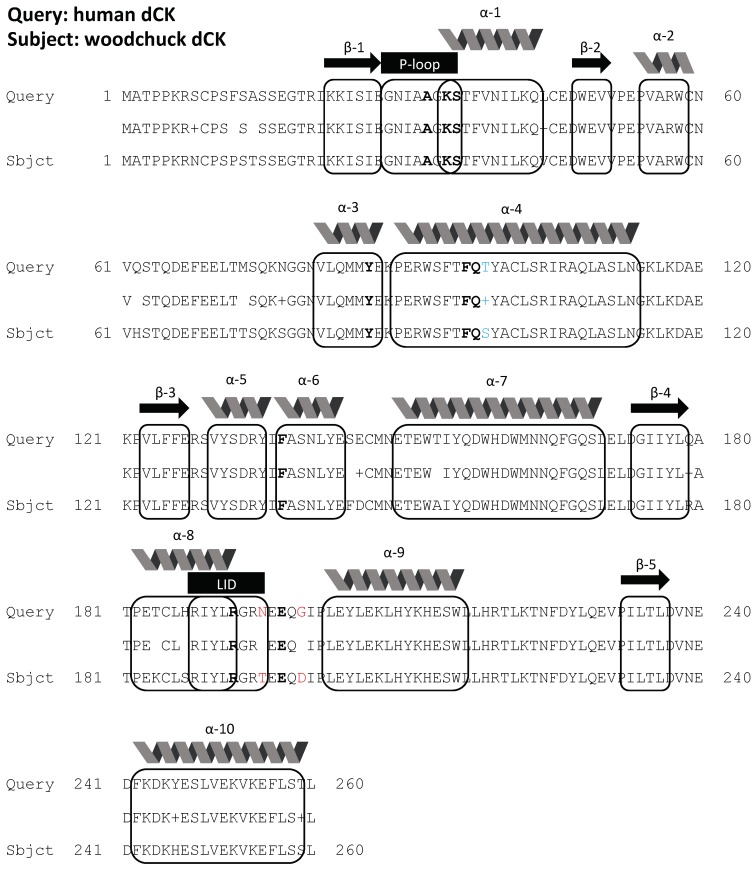
The amino acid sequence alignment between human dCK and marmot dCK, revealing a 96% homology. β-sheets 1–5 and α-helices (1–10) are indicated above boxed amino acid sequences. Sequences in bold are conserved residues between the two species for substrate binding. Residues in blue represent sequence differences between compatible amino acid species located adjacent to conserved substrate binding residues, while red indicates potentially incompatible amino acid variances adjacent to the conserved residues.

**Figure 3:**
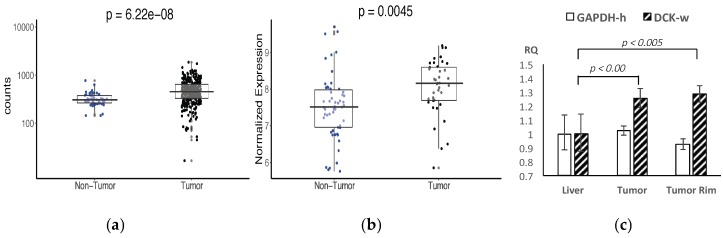
The expression of dCK was found to be higher in hepatocellular carcinoma (HCC) than the surrounding liver tissues from both human (**a**: RNA-seq results) and woodchuck (**b**: Microarray results) data, and verified by PCR with harvested woodchuck tissues (**c**: Using woodchuck-specific primer for dCK).

Figure 2 shows the alignment of dCK protein sequences from human and woodchuck, with a homology of 96% between the two species, and with the key amino acid residues (shown in bold letters) forming the binding pocket for dCK substrates identical between the two. Figure 3a,b shows a higher dCK expression in HCC than the surrounding liver tissues from both human (RNA-seq) and woodchuck (microarray) results. PCR with harvested woodchuck liver tissues using the woodchuck-specific primer for dCK verified the higher expression of dCK in HCC (Figure 3c). Inversely, PNP showed a higher expression, in terms of the base main count in either RNA-seq (human) or microarray (woodchuck) data, in the liver parenchyma than in HCC (Figure S1 in Supplementary Materials) for human and woodchuck, with a 92% homology between the two species. 

Among the nucleoside transporters responsible for cellular CFA uptake, ENT2 and maybe ENT1 contributed to contrast uptake of CFA in HCC (Table 1), in addition to passive diffusion. ENT1 and ENT2 are 90% and 93%, respectively, homologous between the two species, while UGT1A1, UGT1A9, UGT2B4, and UGT2B15, the dominant hepatic enzymes from the UGT family [15], showing a homology of 88%, 88%, 52%, and 86%, respectively. Noticeably, the predicted marmot UGT2B4 sequence is much shorter than the human UGT2B4 sequence, which resulted in many gaps in the alignment between the two species for UGT2B4, while a higher degree of homology (85%) was found between the woodchuck UGT2B4 and human UGT2B15. The FASTA results of PNP, ENT1, ENT2, and the UGTs are included in Supplementary Materials. The expression of all UGTs is significantly higher in the liver parenchyma than in HCC; also see Table 2.

### 2.2. PET Imaging

**Figure 4:**
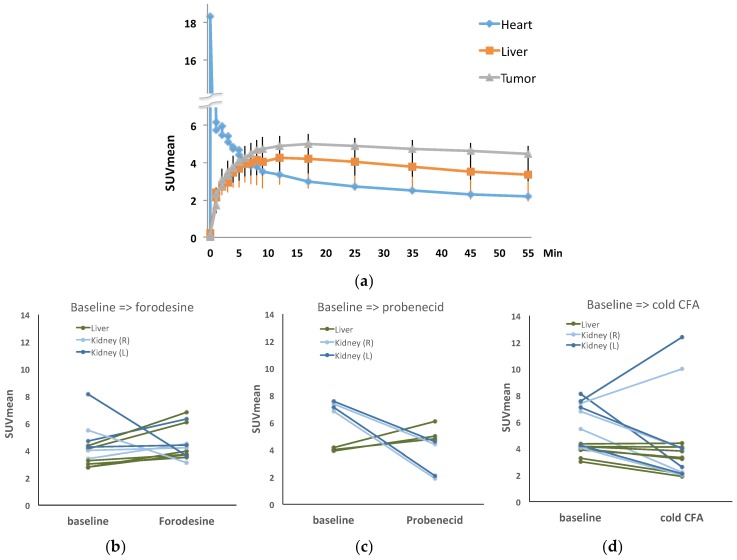
Image-derived quantitation. Time activity curves from the baseline dynamic PET imaging confirmed [^18^F]CFA uptake in both HCC and liver background in the woodchuck model (**a**). Application of the PNP inhibitor, forodesine, did not reduce the background uptake, although some kidney uptakes were reduced, as it increased liver uptake by 11.6–36.3% (**b**). Application of probenecid also increased liver background uptake by 16.6–32.1%, while the kidney uptake was reduced (**c**). Application of cold CFA decreased liver background uptake (**d**). Note: Standardized uptake values (SUVs) were calculated at 55 min (average within 50–60 min) into [^18^F]CFA PET scan.

**Figure 5:**
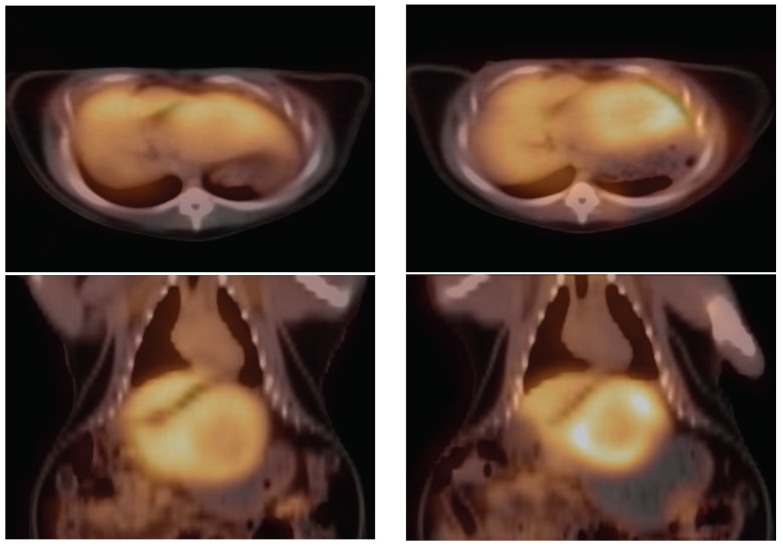
Comparing [^18^F]CFA scans. PET/CT overlays: Upper, trans-axial; lower, coronal. Right, baseline; left, application of cold CFA prior to [^18^F]CFA injection, with heightened uptake in the tumor rim comparing to the baseline.

**Figure 6:**
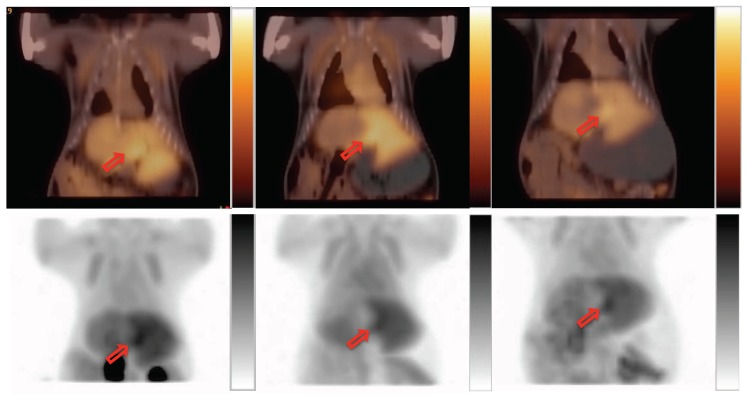
Three-way comparison of [^18^F]CFA uptake in tumor (red arrow) and liver. From the left: Baseline, post UGT inhibitor probenecid application, and after cold CFA application. In each column, the upper panel is PET/CT overlay, while the lower panel is the maximal intensity projection (MIP).

Radiosynthesis was automated using the ELIXYS module, which produced [^18^F]CFA with a consistent radiochemical yield >10%, radiochemical purity >99%, and chemical purity >90%. The regional activity curves from the baseline dynamic PET imaging confirmed [^18^F]CFA uptake in both HCC and liver background in the woodchuck model (Figure 4a). When modifiers were applied before [^18^F]CFA injection, only the plateaued level of [^18^F]CFA uptake (50–60 min post-injection) in the liver changes, while liver tumor uptake of [^18^F]CFA did not change considerably in comparison to the baseline scan. Application of the PNP inhibitor, forodesine (Figure 4b), did not reduce the background level. Instead, it increased the background level by 11.6–36.3%. Cold CFA application (Figure 4d) before hot [^18^F]CFA injection was the only intervention that reduced the liver background uptake of [^18^F]CFA to render a better contrast uptake of [^18^F]CFA in HCC. Figure 5 shows such a comparison between the baseline and the application of cold CFA. Application of probenecid (Figure 4c) increased the liver background level by 16.6–32.1%. Figure 6 shows an example of a three-way comparison (baseline, UGT inhibitor probenecid, and cold CFA,). In Figure 6 (middle column), the uptake of kidneys was reduced with the application of probenecid. Similar results from other animals can be found in Supplementary Materials, in which Figure S2 presents another animal with the same three-way comparison as in Figure 6, but with concentrated signal in gallbladder upon cold CFA application; while Figure S3 presents two more animals with a different three-way comparison of baseline, PNP inhibitor forodesine, and cold CFA applications. 

### 2.3. Validation

#### 2.3.1. Enzymatic Assay for dCK 

**Figure 7:**
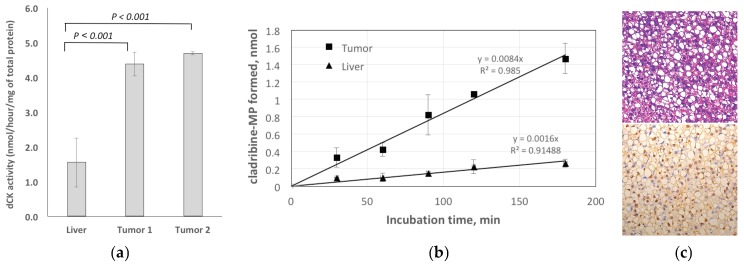
Validation: (**a**) The enzymatic assay of dCK showed that the average dCK activity in HCC samples was 2.8–3.0-fold higher than in normal liver when normalized to the total protein contents. (**b**) Time dependence of the dCK reaction, showing the amount of CdAMP formed by dCK. (**c**) H&E staining (upper panel) revealed mostly moderately-differentiated HCC with a noticeable level of inflammation and steatosis, and positive PCNA (nuclear) staining (lower panel) indicated the proliferative nature of the tumor with [^18^F]CFA uptake.

The enzymatic activity of dCK in liver and two tumor tissue samples was measured. After 30 min reaction, the average dCK activity in HCC samples was 2.8–3.0-fold higher than in normal liver (Figure 7a). The dCK activity was 1.55 nmol/hour/μg of total protein for liver tissue, while HCC extracts showed 4.38 and 4.68 nmol/hour/μg of total protein dCK activity for tumor samples. Figure 7b shows the time-dependence of the dCK assay results for both tissue extracts. In both cases, the reactions were linear for 3 hours, while the formation of cladribine-5-monophosphate (CdAMP) from cladribine (CdA) by HCC extract was higher in comparison with normal liver extract. At 30 min time point, the difference was 3-fold, whereas at 180 min reaction, the variance became 6-fold.

#### 2.3.2. Histology

Figure 7c shows H&E staining (10X) and PCNA staining (20X) in HCC. The tumors were assessed as mostly moderately-differentiated with a noticeable level of inflammation and steatosis. PCNA (nuclear) staining was positive indicating the proliferative nature of the tumor with radiotracer uptake.

## 3. Discussion

Currently, there is no “good” radiotracer for PET imaging to supplement standard radiological imaging of HCC. The commonly used glucose analog, 2-[^18^F]fluoro-2-deoxy-D-glucose (FDG), does not show uptake in some HCCs, leading to a high false negative rate [17]. [^18^F]FAC targets the same dCK, and displays a low hepatic background uptake during PET imaging [18]. Anecdote evidence suggested deamination of D-FAC by hepatic cytidine deaminase, while L-FAC, which is resistant to hepatic deamination, showed a higher liver background [18]. The long radio-synthesis time needed for both D- and L-FAC, even automated, posed a logistic challenge for their routine clinical use [19]. Due to its more straightforward radiolabeling procedure, comparing to that of FAC, [^18^F]CFA was adopted for investigation as an alternative to FAC for PET imaging of dCK-dependent proliferation [3], and here, for HCC in this study. A high degree of homology in protein sequence of dCK between human and woodchuck (Figure 2) ensured targeting of CFA and [^18^F]CFA to dCK in both species.

The expression of dCK is higher in some HCCs than in the surrounding liver parenchyma, as revealed by our bioinformatics analyses (Figure 3) and verified by PCR (Figure 3c) and enzymatic assays (Figure 7a), which makes it a potentially viable imaging marker for tracking dCK-dependent proliferation (Figure 7c). Importantly, dCK is a pyrimidine metabolism enzyme essential to the activity of several anti-cancer pro-drugs such as gemcitabine and decitabine. The level of dCK can be imaged potentially as a biomarker for evaluating cancer treatment with these drugs. For some HCCs with lower activity of dCK to begin with, interim treatment with other drugs that interact with pyrimidine metabolism, or with uridine-cytidine kinase 2 (UCK2)-dependent drugs, might at least partially restore dCK activity and possibly increase CFA uptake in those cancers with initially low CFA uptake. [^18^F]CFA can thus be used to detect this switch to facilitate treatment decision-making.

CFA is a slightly lipophilic drug, entering into the cells by passive diffusion across lipid membranes, and via ENT1, ENT2, CNT2, and CNT3 [20], of which human ENT2 [16] and maybe ENT1 seemed to be the nucleoside transporter that is up-regulated slightly in HCC [5], and is homologous to the woodchuck counterpart. High liver background uptake in humans and woodchucks posed a problem for the direct use of [^18^F]CFA for liver or liver cancer imaging, which is not a problem with rodents, as a higher concentration of circulating deoxycytidine in murine, 2–3 orders higher than humans, helped to saturate the metabolic enzymes and lower the background uptake of CFA in the mouse liver [3]. Based on this, the cold CFA was applied, before [^18^F]CFA injection and PET imaging, at 4.2 mg/kg to the woodchuck models, a dose converted from the equivalent of the high (human) pediatric dose of 52 mg/m^2^ [21]. This effectively decreased [^18^F]CFA uptake in the liver background in HCC, as shown in Figure 4, leading to better contrast tumor uptake of [^18^F]CFA, shown in Figure 5 and Figure 6. It is known that dCK is not rate limiting for CFA [22] and a higher dCK activity in HCC than in the surrounding liver tissues could make it less affected by the saturating cold CFA to render a better contrast uptake in HCC. 

Substitution of a fluorine atom at the arabinosyl configuration at the 2’-position of the carbohydrate decreased the susceptibility of CFA to phosphorolytic cleavage by PNP. However, the high expression PNP in the liver parenchyma, as confirmed by the bioinformatics (Figure S1), prompted the test of a PNP inhibitor, forodesine (BCX-1777), with the hope that inhibition of PNP would prevent the formation of [^18^F]arabinofuranose and hepatic accumulation of the radio-metabolites, and thus lower the liver background signal associated with [^18^F]CFA. The PNP inhibitor forodesine at 6.0 mg/kg applied to the woodchuck models, a dose converted from the equivalent high human dose of 80 mg/m^2^ [23], failed to reduce the liver background uptake of [^18^F]CFA. PNP is also homologous between human and woodchuck, and works in both ways, as shown in Figure S1. It is possible that PNP was initially working, mainly in the inverse direction to prevent hydrolysis of CFA in liver microenvironment, when the application of forodesine inhibits this to increase the forward cleavage, leading to more hydrolysis and a possible increased liver uptake of [^18^F]CFA. 

Probenecid is an FDA-approved drug for the treatment of gout and hyperuricemia by renal clearance of uric acid, but it was tested in this study for its inhibitory effect on hepatic glucuronidation [13]. Due to its remarkable safety profile, a very high dose of probenecid was applied orally before [^18^F]CFA injection and PET imaging. The timing of oral administration is based on peak plasma concentration in humans after oral uptake. The results showed the opposite effect, as the application of probenecid increased the liver background uptake of [^18^F]CFA (Figure 6). Bioinformatics data showed active UGT expression in liver parenchyma (Table 2), and the UGTs were mostly homologous between human and woodchuck. Probenecid broadly inhibited many isoforms of UGT in a non-specific manner by inhibiting the clearance of glucuronides [24], which accumulated as retention to potentially explain the higher liver background uptake after probenecid application, while the uptake in the kidneys was, nevertheless, cleared out as expected.

Only cold CFA reduced the liver background uptake of [^18^F]CFA, while the mechanism for the high hepatic background uptake of [^18^F]CFA remains to be illustrated. CFA was conditionally approved due to its toxicity profile. Since CFA will not be applied daily or consecutively, as in treatment, but will only be administered prior to [^18^F]CFA injection for clinical PET imaging, it is hopeful for this cold/hot CFA combination to be a viable strategy for imaging dCK-dependent proliferation in liver cancers. Although the equivalent of a high human dose was tested in this study, further titration for the optimized dose can be sought in future clinical studies. More importantly, the timing of cold CFA application before [^18^F]CFA injection can also be investigated in the future for optimized tumor-to-liver uptake ratio.

## 4. Materials and Methods

### 4.1. Animal Models

Woodchucks weighted 8–10 lbs (averaged 3.5 kg) and aged 2–3 years old were scanned with ultrasound at Roswell Park Comprehensive Cancer Center (Buffalo, NY, USA) and selected for shipment to Case Western Reserve University (Cleveland, OH, USA) when the liver nodules were larger than 20 mm in size. A venous access port (SAI Infusion Technologies; Elgin, IL, USA) was surgically implanted in each animal to facilitate radiotracer injections for all PET scans. The port was flushed regularly with heparinized saline. The food was taken away 4–5 hours before each PET imaging session, while drinking water was always kept. All procedures were approved by the Institutional Animal Care and Use Committee of the University (IACUC#2014-0085).

### 4.2. Bioinformatics

The expression of the key genes involved in the transport and metabolism of CFA: dCK, PNP, ENTs and CNTs, and UGTs between liver tumors and non-tumor liver tissues were tallied from the databases. The human data came from TCGA, and the woodchuck data from the customized microarray (see below). Homology of amino acid sequences between human (*Homo sapiens*) and woodchuck (*marmot*) proteins was determined by using the Protein Basic Local Alignment Search Tool (BLAST) (https://blast.ncbi.nlm.nih.gov/Blast.cgi). Specifically, the homology between the two species was searched for dCK (NCBI Reference Sequence: NP_000779.1 (*Homo sapiens*) and NCBI Reference Sequence: XP_015360791.1 (*Marmota marmota marmot*)). Similarly, the homology between the two species was also searched for PNP, ENT1, ENT2, UGT1A1, UGT1A9, UGT2B4, and UGT2B15, and described in Supplementary Materials.

#### 4.2.1. Human Data

TCGA data were downloaded from the public TCGA Liver Hepatocellular Carcinoma database (TCGA-LIHC). The data included a total of 371 RNA-seq gene expression results collected from both tumor and non-tumor human tissues. The TCGA-LIHC data were all sequenced using Illumina platforms and available as “raw” (i.e., un-normalized) read counts for each gene for each sample. Data were then reformatted and sent through the DESeq2 workflow in R [25], including a pre-filtering step, where genes with very low gene counts were excluded, a normalization step using DESeq2’s median of ratios suitable for comparing genes across samples, calculating the log2 fold change between non-tumor and tumor samples for each gene, and determining the significantly and differentially expressed genes between non-tumor and tumor genes with a Wald test. The final results yielded the base mean for each gene, the log2 fold change, the standard error for the fold change, the Wald test statistic, and the raw and adjusted *p*-value for multiple testing correction using FDR. Genes from the human data were not directly compared to the woodchuck data. Differentially expressed genes were only compared within species independently. 

#### 4.2.2. Woodchuck Data

The woodchuck data were collected from the NCBI Gene Expression Omnibus (accession number GSE36545 and BioProject PRJNA155585). The data included 102 samples from 13 animals, with a total of 42 tumor samples and 60 non-tumor samples (GSM896624-GSM896725) [26]. The data obtained were from a custom NimbleGen Woodchuck Gene Expression HX3 Microarray. The downloaded data were normalized gene expression data for each sample, as previously reported [26]. Nine outlying samples (from three animals, triplicated) were identified with principal component analysis and subsequently excluded from the analysis. Log2 fold change and *t*-tests were then calculated to compare the gene expression between non-tumor and tumor samples. All resulting *p*-values were FDR-corrected to adjust for multiple testing. Genes from the woodchuck data were not directly compared to the human data. Differentially expressed genes were only compared within species independently, and therefore, any technical bias inherent in microarray and high-throughput sequencing is consistent in the respective comparison.

### 4.3. PET Imaging

#### 4.3.1. Radiotracer

[^18^F]CFA was synthesized automatically with the ELIXYS module (SOFIE, Culver City, CA, USA) using the sequence provided by the vendor. The eluent solution to elute fluoride off of ABX QMA was prepared by adding 7.5 µL of 0.1 mg/µL K_2_CO_3_ solution, 44.9 µL of 0.1 mg/µL Kryptofix 2.2.2. solution, 348 µL of water, and 600 µL of acetonitrile directly into a reagent vial. The 1-pot synthesis of [^18^F]CFA was purified on Kinetex EVO column with the following elution conditions: Isocratic, 95% Acetonitrile 5% 25 mM NH_4_OAc (aq); 1 mL/ min. 

#### 4.3.2. Imaging Experiment

The spontaneously developed HCC in the woodchucks after chronic hepatitis was used for PET imaging experiments. For the baseline scan, the animal was placed prone in the clinical Ingenuity PET/CT scanner (Philips, Cleveland, OH) and under 3% isoflurane gas anesthesia throughout the scan. After a low-dose CT scan, 37~56 MBq (1.0~1.5 mCi) [^18^F]CFA was injected intravenously (i.v.) through the implanted venous access port and followed by a dynamic PET scan of 60 min in list mode on the clinical PET/CT system, as the woodchucks with an average weight of 3.5 kg did not fit into the microPET. PET acquisition was re-binned into a total of 21 frames: 10 × 30 seconds, 5 × 1-min, 2 × 5-min, and 4 × 10 min frames, respectively. After the baseline scan, additional dynamic PET scans with [^18^F]CFA were performed identically with the same animal after the application of one of the modifiers. For PNP inhibition, forodesine at 6.0 mg/kg i.v. 5 min before [^18^F]CFA injection; for inhibiting hepatic glucuronidation, probenecid at 0.25 g/kg orally 2 h before [^18^F]CFA injection; for enzymatic saturation, cold CFA applied at 4.2 mg/kg 10 min before the hot [^18^F]CFA injection. Three animals were each scanned three times in the sequence of baseline, fordesine, and cold CFA applications prior to [^18^F]CFA injection; and two animals were scanned three times in the sequence of baseline, probenecid, and cold CFA applications. All three scans for each animal were scheduled as close as possible, giving the animals anesthetic recovery between the scans, within two weeks. After the final scan, the animals were euthanized for tissue harvesting, including the tumor and the matched liver for each animal. Some of the samples were fresh-frozen immediately for later use in PCR or enzymatic assay, while others were fixed with formaldehyde for histology.

#### 4.3.3. Data Analysis

Standardized uptake value (SUV) [27] was calculated for regions of interest (ROIs) defined over focal uptakes of CFA, as well as a nearby ROI for liver background away from focal uptakes, similar to that for FDG uptake [28], and time activity curves in the unit of SUV were generated for these ROIs. Comparison of SUVs at 55 min post-injection of [^18^F]CFA was made between the baseline and post-modifier scans to evaluate the reduction of liver background uptake of the [^18^F]CFA from the baseline due to the application of the modifiers (forodesine, probenecid, cold CFA).

### 4.4. Validation

#### 4.4.1. qRT-PCR 

The primers for qRT-PCR were designed using The Custom TaqMan^®^ Assay Design Tool based on marmot mRNA sequences for required genes: dCK (custom TaqMan gene expression assay AP7DRFY based on XM_015505305.1mRNA sequence), and endogenous control gene GAPDH (assay AP2W9P7 based on XM_015500718.1 mRNA sequence). RNA was extracted from tissue using an Qiagen miRNeasy Mini Kit (Cat. No.217004, Qiagen), according to manufacturer’s instructions. Total RNA (0.1 µg for each reaction) was used to generate complementary DNA (cDNA) with the High-Capacity RNA-to-cDNA Kit (Cat. No. 4387406; Applied Biosystems). qRT-PCR was performed on a StepOne Plus real-time thermocycler with 1.33 mL of cDNA for each reaction and the TaqMan Universal master Mix II, with UNG (Cat. No. 4440038; Applied Biosystems). Expression data were obtained for each gene from each sample as threshold cycle (Ct). ΔCt was calculated as the Ct of endogenous control gene minus the Ct of the gene of interest. ΔΔCt was then calculated as the ΔCt of the reference sample minus the ΔCt of another sample. This set the ΔΔCt of the reference sample to 0. The relative quantification of gene expression (RQ) was calculated as 2^−(ΔΔCt)^. This yielded an RQ for the reference sample as 1. Samples with more transcripts than the reference sample will have negative ΔΔCt scores and larger RQ values.

#### 4.4.2. Enzymatic Assay for dCK 

The dCK assay follow the method described by Bierau et al., with a few modifications [29]. The frozen animal tissue samples were homogenized using the pestle mortar method, and added into the lysis buffer containing 50 mM Tris–HCl pH 7.4, 200 mM NaCl, 0.1% triton x-100, 5 mM DTT (dithiothreitol), 2 mM PMSF (phenylmethylsulphonylfluoride), and protease inhibitor cocktail. The homogenized samples were passed 2–3 times through 25 G needle, after that the samples were incubated for 30 min on ice and were then centrifuged for 15 min at 20,000 × *g* at 4 °C to remove any remaining insoluble material. The supernatant was used for dCK activity assays, while the pellet was discarded. Protein content was determined using Pierce 660 nm protein assay (Thermo Scientific™, #22660, Grand Island, NY, USA), according to manufacturer’s instructions. The reaction solution consisted of 1 mM CdA, 10 mM ATP, 10 mM MgCl_2_, 200 mM NaCl, 20 mM NaF, 5 mM DTT in 10 mM Tris–HCl pH 7.4. In a typical assay, to 25 µL of the reaction solution, 25 µL of tissue homogenate was added. The reaction started by putting the tube containing the reaction mix in a water bath at 37 °C. After an appropriate incubation time (30–180 min) at 37 °C, the reaction was terminated by placing the reaction tube on ice and adding 50 μL of ice-cold methanol, causing precipitation of the protein. After 10 min of incubation on ice, the samples were either directly prepared for HPLC analysis or stored at −20 °C until analysis. Immediately after mixing the cell extract with the reaction solution, reaction blanks were created by performing the methanol-precipitation. Prior to HPLC analysis, the samples were centrifuged for 5 min at 10,000 × *g* at 4 °C and diluted two-fold with a 50 mM NH_4_H_2_PO_4_ (pH not adjusted) solution.

Reaction products were quantified by reversed phase HPLC at ambient temperature using a 100 × 4.6 mm Luna 5 µm C18(2) column (Phenomenex, Green Land, NY, USA) at a flow rate of 1 ml/min, using a gradient of 50 mM NH_4_H_2_PO_4_ (buffer A, pH unadjusted) and 50% (v/v) methanol in 50 mM NH_4_H_2_PO_4_ (buffer B). The gradient used was: 3 min at 90% buffer A, in 6 min to 50% buffer A, hold for 4 min, then in 3 min return to 90% buffer A. Detection of CdA and CdAMP was performed at 265 nm. CdA and CdAMP concentrations were calculated using pure compounds as standards. The enzymatic was calculated from the product formed in the reaction and expressed as nanomoles of CdAMP composed per hour and milligram of total protein.

#### 4.4.3. Histology

For tissues fixed in formalin, H&E staining was performed, as well as immunohistochemical (IHC) staining for proliferative status using the anti-PCNA antibody [30] (PC10, from Abcam, Cambridge, MA, USA). The liver pathologist evaluated the liver tumor based on the H&E staining.

## 5. Conclusions

The inhibition of either PNP or UGTs failed to reduce the liver background uptake of [^18^F]CFA. The cold CFA reduced the liver background uptake of [^18^F]CFA, while the mechanism for the high hepatic background uptake of CFA remains to be illustrated. Combining cold CFA with [^18^F]CFA for clinical PET imaging of dCK-dependent proliferation in liver cancers seems to be a viable strategy worthy of further clinical investigation.

## Figures and Tables

**Table 1:** Log2 fold change (log2FC) of nucleoside transporter expression between HCC and liver.

Transporters	RNA-Seq (Human)		Microarray (Woodchuck)	
	log2FC	adj. *p*-Value	log2FC	adj. *p*-Value
ENT1 (SLC29A1) ^1^	0.73183556	8.55E–07	N/A	N/A
ENT2 (SLC29A2)	0.97080408	2.76E–10	0.3009289	4.31E–02
CNT2 (SLC28A2) ^2^	0.04387712	*9.06E–01*	1.0875916	5.49E–08
CNT3 (SLC28A3)	−1.0067800	2.21E–02	−1.086378	2.14E–07

^1^ The entry of ENT1 (SLC29A1) is missing from the annotated woodchuck (customized) microarray data, while the results of ENT2 from human and woodchuck point to the same direction of slightly higher in HCC in comparison with the surrounding liver tissues. ^2^ For CNT2 (SLC28A2), there is no differential expression between HCC and the surrounding liver (adjusted *p* = 0.906) from the human results, while both human and woodchuck data showed a down-regulation (more than two-fold) of CNT3 in HCC, comparing to the surrounding liver tissues.

**Table 2:** Hepatic expression (the base main counts) of UGTs, comparing to HCC.

UGTs	RNA-Seq (Human)			Microarray (Woodchuck)		
	liver	HCC	adj. *p*-Value	liver	HCC	*adj. p*-Value
UGT1A1 ^1^	8801.711867	3533.469471	1.45163E–05	13.62426111	13.13745641	4.23181E–06
UGT1A9 ^2^	2466.358055	1734.905503	0.142498018	13.55701481	13.50425897	0.673895776
UGT2B4 ^3^	45576.22225	41455.97744	0.589224659	13.79634444	13.15938462	1.90991E–06
UGT2B15 ^4^	19323.67142	12831.68579	0.035838247	11.81149	10.58412	3.53358E–15

^1^ 37% of human UGT1A1 is localized in the liver; ^2^ 31% of human UGT1A9 is in the liver; ^3^ 59% of human UGT2B4 is localized in the liver, but woodchuck UGT2B4 is more homologous to human UGT2B15 (see the FASTA results in the Supplemental Materials); ^4^ 29% of human UGT2B15 is in the liver [16].

## Supplementary Materials

The following are available online at https://www.mdpi.com/2072-6694/11/11/1748/s1, Figure S1: PNP levels in the liver, FASTA (alignment) results between human and woodchucks for PNP, ENT1, ENT2, UGT1A1, UGT1A9, UGT2B4, and UGT2B15, Figure S2: Three-way comparison of [18F]CFA uptake in tumor (red arrow) and liver. From the left: baseline, post UGT inhibitor probenecid application, and after cold CFA application. In each column, the upper panel is PET/CT overlay while the lower panel is the maximal intensity projection (MIP), Figure S3: Three-way comparison of [18F]CFA uptake in tumor (red arrow) and liver. From the left: baseline, post PNP inhibitor forodesine application, and after cold CFA application. In each column, the upper panel is PET/CT overlay while the lower panel is the maximal intensity projection (MIP).
