# Supplementary material for: [18F] Clofarabine for PET Imaging of Hepatocellular Carcinoma
Source: Cancers (Basel). 2019 Nov 7;11(11):1748. doi: 10.3390/cancers11111748 (PMC6896045; doi:10.3390/cancers11111748)
Supplement: Supplementary file 1 [file cancers-11-01748-s001.pdf]

cancers

MDPI

Supplementary Materials: [^{18}F] Clofarabine for PET Imaging of Hepatocellular Carcinoma

Purine-nucleoside: orthophosphate ribosyltransferase (EC 2.4.2.1) [1,2]

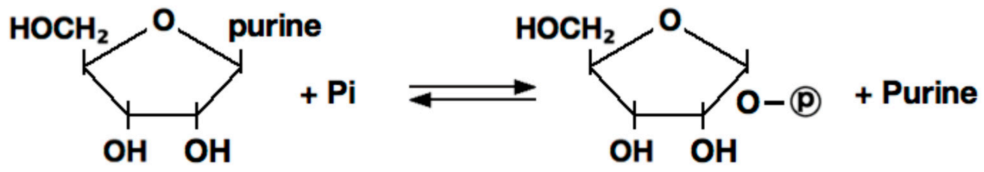

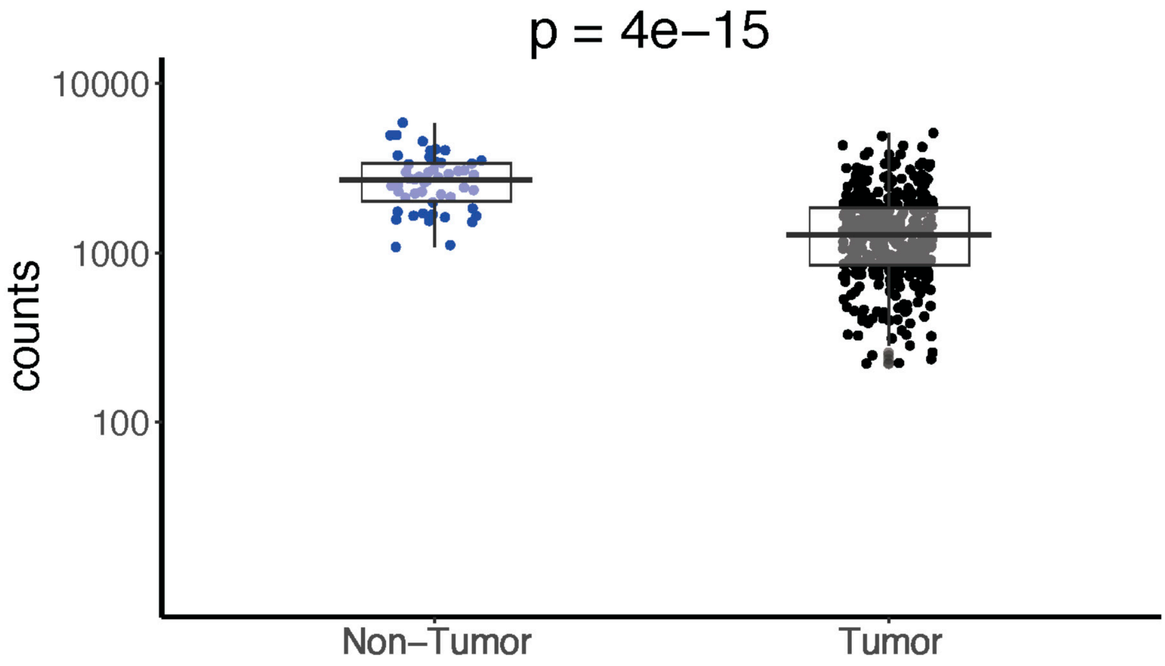

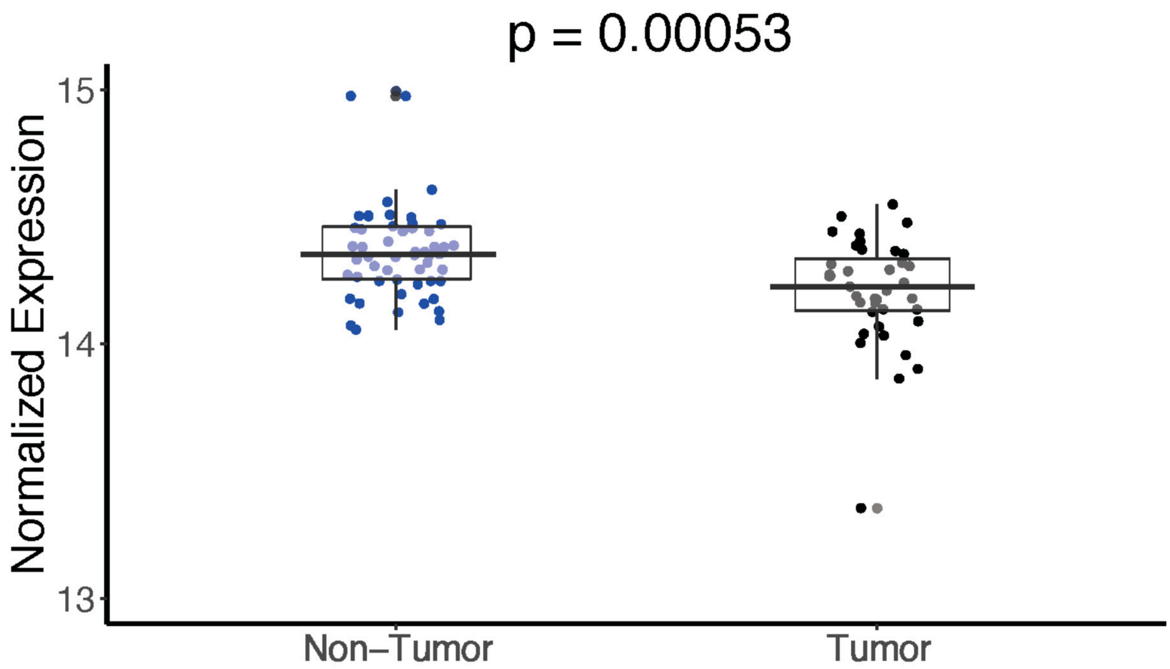

Figure S1. PNP levels in the liver. PNP has a significantly higher expression, in terms of the base main counts in either RNA-seq (human) or microarray (woodchuck) data, in the liver parenchyma than in HCC for human and woodchuck. The enzyme works reversibly in both directions.

The FASTA results

1. PNP alignment between

Query: NCBI Reference Sequence: NP_000261.2; purine nucleoside phosphorylase [Homo sapiens]

Sbjct: NCBI Reference Sequence: XP_015336353.1; PREDICTED: purine nucleoside phosphorylase [Marmota marmota marmota]

```
Identities        Positives         Gaps
251/288(87%)      265/288(92%)      0/288(0%)

Query  1    MENGYTYEDYKNTAEWLLSHTKHRPQVAIICGSGLGGLTDKLTQAQIFDYGEIPNFPRST  60
            MEN +TYEDY+ TAEWLLS TKHRPQVA+ICGSGLGGLT+KLT+AQ FDY EIPNFP+ST
Sbjct  1    MENEFTYEDYQKTAEWLLSRTKHRPQVAVICGSGLGGLTNKLTEAQFFDYSEIPNFPQST  60

Query  61   VPGHAGRLVFGFLNGRACVMMQGRFHMYEGYPLWKVTFPVRVFHLLGVDTLVVTNAAGGL  120
            VPGHAGRLVFGFLN +ACVMMQGRFH YEGYPLWKVTFPVRVF LLGVDTLVVTNAAGGL
Sbjct  61   VPGHAGRLVFGFLNDKACVMMQGRFHTYEGYPLWKVTFPVRVFRLLGVDTLVVTNAAGGL  120

Query  121  NPKFEVGDIMLIRDHINLPGFSGQNPLRGPNDERFGDRFPAMSDAYDRTMRQRALSTWKQ  180
            NP F+VGDIMLIRDHINLPG  GQNPL+GPNDERFG RFPAMSDAYDR MR++ALSTWK
Sbjct  121  NPNFQVGDIMLIRDHINLPGLGGQNPLKGPNDERFGVRFPAMSDAYDRNMREKALSTWKL  180

Query  181  MGEQRELQEGTYVMVAGPSFETVAECRVLQKLGADAVGMSTVPEVIVARHCGLRVFGFSL  240
            MGE RELQEGTYVMVAGPSFETVAE R+LQ+LGADAVGMSTVPEVIVARHCGLRVFGFSL
Sbjct  181  MGENRELQEGTYVMVAGPSFETVAESRLLQQLGADAVGMSTVPEVIVARHCGLRVFGFSL  240

Query  241  ITNKVIMDYESLEKANHEEVLAAGKQAAQKLEQFVSILMASIPLPDKA  288
            ITNKVIMDYESLEK  HEEVL  GKQAAQKLE+FVSILMASIP  D A
Sbjct  241  ITNKVIMDYESLEKTTHEEVLETGKQAAQKLEKFVSILMASIPPRDNA  288
```

2. ENT1 alignment between

```
Query: UniProtKB/Swiss-Prot: Q99808.3; S29A1_HUMAN RecName: Full=Equilibrative
nucleoside transporter 1; AltName: Full=Equilibrative nitrobenzylmercaptopurine
riboside-sensitive nucleoside transporter; Short=Equilibrative NBMPR-sensitive
nucleoside transporter; AltName: Full=Nucleoside transporter, es-type; AltName:
Full=Solute carrier family 29 member 1

Sbjct: NCBI Reference Sequence: XP_015342854.1; PREDICTED: equilibrative nucleoside
transporter 1 [Marmota marmota marmota]
222


Identities        Positives         Gaps
404/464(87%)      421/464(90%)      9/464(1%)

Query  2     TTSHQPQDRYKAVWLIFFMLGLGTLLPWNFFMTATQYFTNRLDMSQNVSLVTAELSKDAQ  61
             TTSHQPQDRYKAVWLIFFMLGLGTLLPWNFFMTAT+YFTNRLDMSQNVSL  A LSKD Q
Sbjct  77    TTSHQPQDRYKAVWLIFFMLGLGTLLPWNFFMTATKYFTNRLDMSQNVSLNAAVLSKDIQ  136

Query  62    ASAAPAAPLPERNSLSAIFNNVMTLCAMLPLLLFTYLNSFLHQRIPQSVRILGSLVAILL  121
              A P APLPE+ SLSAIFNNVMTLCAMLPLLLF+ LNSFLHQRIPQSVRILGSLVAILL
Sbjct  137   DPAVPTAPLPEQRSLSAIFNNVMTLCAMLPLLLFSCLNSFLHQRIPQSVRILGSLVAILL  196

Query  122   VFLITAILVKVQLDALPFFVITMIKIVLINSFGAILQGSLFGLAGLLPASYTAPIMSGQG  181
             VFL+TAILVKVQLDALPFFV TMIKI+LINSFGAILQGSLFGLAGLLPA+YTAPIMSGQG
Sbjct  197   VFLVTAILVKVQLDALPFFVFTMIKIILINSFGAILQGSLFGLAGLLPANYTAPIMSGQG  256

Query  182   LAGFFASVAMICAIASGSELSESAFGYFITACAVIILTIICYLGLPRLEFYRYYQQLKLE  241
             LAG FASVAMICAIASGSELSESAFGYFITAC V+IL IICYLGLPRLEFYRYYQQLKLE
Sbjct  257   LAGLFASVAMICAIASGSELSESAFGYFITACVVVILAIICYLGLPRLEFYRYYQQLKLE  316

Query  242   GPGEQETKLDLISK---------GEEPRAGKEESGVSVSNSQPTNESHSIKAILKNISVL  292
             GPGEQETKLDLISK         GEEPRA KEESGVS  NSQ T+ + SI+AILKNISVL
Sbjct  317   GPGEQETKLDLISKDSSTTCHLPGEEPRASKEESGVSTPNSQATSRNPSIRAILKNISVL  376

Query  293   AFSVCFIFTITIGMFPAVTVEVKSSIAGSSTWERYFIPVSCFLTFNIFDWLGRSLTAVFM  352
             A SVC  FT+TIG+FPAVT EVKSSIAG+S W  YFIPVSCFLTFNIFDWLGRSLTAVFM
Sbjct  377   ALSVCCNFTVTIGLFPAVTAEVKSSIAGTSAWGDYFIPVSCFLTFNIFDWLGRSLTAVFM  436

Query  353   WPGKDSRWLPSLVLARLVFVPLLLLCNIKPRRYLTVVFEHDAWFIFFMAAFAFSNGYLAS  412
             WPGKDS WLPSLV+ARLVFVPLLLLCN+ PRRYL VVF+HDAW+IFFMAAFAFSNGYLAS
Sbjct  437   WPGKDSCWLPSLVVARLVFVPLLLLCNVHPRRYLAVVFKHDAWYIFFMAAFAFSNGYLAS  496

Query  413   LCMCFGPKKVKPAEAETAGAIMAFFLCLGLALGAVFSFLFRAIV  456
             LCMCFGPKKVKPAEAETAGAIM FFL LGLALGAV SFL RAIV
Sbjct  497   LCMCFGPKKVKPAEAETAGAIMTFFLSLGLALGAVLSFLLRAIV  540
```

3. ENT2 alignment between

```
Query: NCBI Reference Sequence: NP_001523.2 equilibrative nucleoside transporter 2
isoform a; SLC29A2 [Homo sapiens]

Sbjct: NCBI Reference Sequence: XP_015358637.1 PREDICTED: equilibrative nucleoside
transporter 2 isoform X1 [Marmota marmota marmota]


Identities       Positives        Gaps
308/339(91%)     318/339(93%)     3/339(0%)

Query  121   MSPGPFFSITMASVCFINSFSAVLQGSLFGQLGTMPSTYSTLFLSGQGLAGIFAALAMLL  180
             MSPGPFFSITM SV FINSF AVLQGSLFGQLGTMPSTYSTLFLSGQGLAGIFAALAML+
Sbjct  1     MSPGPFFSITMVSVWFINSFCAVLQGSLFGQLGTMPSTYSTLFLSGQGLAGIFAALAMLM  60

Query  181   SMASGVDAETSALGYFITPCVGILMSIVCYLSLPHLKFARYYLANKSSQAQAQELETKAE  240
             SMASGVDA+TSALGYFITPCVGILMSIVCYLSLPHL+FARYYLA K S++   ELETKAE
Sbjct  61    SMASGVDAQTSALGYFITPCVGILMSIVCYLSLPHLEFARYYLAKKPSRSPTHELETKAE  120

Query  241   LLQSDE-NGIPSSPQKVALTLDLDLEKEPESEPDEP--QKPGKPSVFTVFQKIWLTALCL  297
             LLQ+DE NGIPSSPQ   LTLDLD EKEPE EP+    QK  KPSVF VFQKIWLTALCL
Sbjct  121   LLQADEKNGIPSSPQVATLTLDLDPEKEPELEPELDEPQKLEKPSVFIVFQKIWLTALCL  180

Query  298   VLVFTVTLSVFPAITAMVTSSTSPGKWSQFFNPICCFLLFNIMDWLGRSLTSYFLWPDED  357
             VLVFTVTLSVFPAITAMVTSSTSPGKWSQFFNPICCFLLFN+MDWLGRSLTSYFLWPDED
Sbjct  181   VLVFTVTLSVFPAITAMVTSSTSPGKWSQFFNPICCFLLFNVMDWLGRSLTSYFLWPDED  240

Query  358   SRLLPLLVCLRFLFVPLFMLCHVPQRSRLPILFPQDAYFITFMLLFAVSNGYLVSLTMCL  417
             SRLLPLLVCLRFLFVPLFMLCHVP+R+RLPILFPQDAYFITFMLLFAVSNGYLVSLTMCL
Sbjct  241   SRLLPLLVCLRFLFVPLFMLCHVPERARLPILFPQDAYFITFMLLFAVSNGYLVSLTMCL  300

Query  418   APRQVLPHEREVAGALMTFFLALGLSCGASLSFLFKALL  456
             APRQVLPHEREVAGALMTFFLALGLSCGASLSFLFKALL
Sbjct  301   APRQVLPHEREVAGALMTFFLALGLSCGASLSFLFKALL  339
```

4. UGT1A1 alignment between

```
Query: NCBI Reference Sequence: NP_000454.1; UDP-glucuronosyltransferase 1-1
precursor [Homo sapiens]

Sbjct: NCBI Reference Sequence: XP_015337083.1; PREDICTED: UDP-
glucuronosyltransferase 1-1 isoform X2 [Marmota marmota marmota]


Identities        Positives         Gaps
433/533(81%)      472/533(88%)      0/533(0%)

Query  1    MAVESQGGRPLVLGLLLCVLGPVVSHAGKILLIPVDGSHWLSMLGAIQQLQQRGHEIVVL  60
            MAV SQG   L+LGLLLCVL P +SHA K+L+IPVDGSHWLSM+G IQQLQ+RGHE+VV+
Sbjct  1    MAVVSQGPCTLLLGLLLCVLNPSISHAEKLLIIPVDGSHWLSMVGVIQQLQKRGHEMVVI  60

Query  61   APDASLYIRDGAFYTLKTYPVPFQREDVKESFVSLGHNVFENDSFLQRVIKTYKKIKKDS  120
             P+AS++I++ +FY+LK YPVPFQ+EDV+ SF  LG   FEN  FLQ V K Y+K +KDS
Sbjct  61   TPEASIHIKEASFYSLKKYPVPFQKEDVETSFAELGFYAFENVPFLQGVAKMYEKAQKDS  120

Query  121  AMLLSGCSHLLHNKELMASLAESSFDVMLTDPFLPCSPIVAQYLSLPTVFFLHALPCSLE  180
            A+LLSGCSHLLHNKE MASL ES FD +LTDPFLPC  IVAQYL LP V FL+ALPC L+
Sbjct  121  AVLLSGCSHLLHNKEFMASLVESDFDAVLTDPFLPCGSIVAQYLDLPAVNFLNALPCGLD  180

Query  181  FEATQCPNPFSYVPRPLSSHSDHMTFLQRVKNMLIAFSQNFLCDVVYSPYATLASEFLQR  240
             +ATQCP+P SYVPR LSS+SDHMTFLQRVKNMLIA  +NFLC +VYSPYA LAS+ LQR
Sbjct  181  LKATQCPSPLSYVPRALSSNSDHMTFLQRVKNMLIALLENFLCSMVYSPYAALASQVLQR  240

Query  241  EVTVQDLLSSASVWLFRSDFVKDYPRPIMPNMVFVGGINCLHQNPLSQEFEAYINASGEH  300
            ++T+QDLLSSASVWL R DFVKDYP PIMPNMVFVGGINCLH  P+SQEFEAY+NASGEH
Sbjct  241  DLTLQDLLSSASVWLMRKDFVKDYPMPIMPNMVFVGGINCLHTKPISQEFEAYVNASGEH  300

Query  301  GIVVFSLGSMVSEIPEKKAMAIADALGKIPQTVLWRYTGTRPSNLANNTILVKWLPQNDL  360
            GIVVFSLGSMVSEIPEKKAM IADALG+IPQTVLWRYTGT PSNLA NT LVKWLPQNDL
Sbjct  301  GIVVFSLGSMVSEIPEKKAMEIADALGRIPQTVLWRYTGTPPSNLAKNTKLVKWLPQNDL  360

Query  361  LGHPMTRAFITHAGSHGVYESICNGVPMVMMPLFGDQMDNAKRMETKGAGVTLNVLEMTS  420
            LGHP TRAFITHAGSHGVYE ICNGVPMVMMPLFGDQMDNAKRMET+GAGVTLNVLEMTS
Sbjct  361  LGHPKTRAFITHAGSHGVYEGICNGVPMVMMPLFGDQMDNAKRMETRGAGVTLNVLEMTS  420

Query  421  EDLENALKAVINDKSYKENIMRLSSLHKDRPVEPLDLAVFWVEFVMRHKGAPHLRPAAHD  480
            +DL NALKAVINDKSYKENIMRLS LHKDRP+EPLDLAVFWVEFVMRHKGAPHLRPAAHD
Sbjct  421  DDLANALKAVINDKSYKENIMRLSRLHKDRPIEPLDLAVFWVEFVMRHKGAPHLRPAAHD  480

Query  481  LTWYQYHSLDVIGFLLAVVLTVAFITFKCCAYGYRKCLGKKGRVKKAHKSKTH  533
            LTWYQYHSLDVIGFLLA+VL VAFI FKCCAYG RKC GKKG VKK HKSK H
Sbjct  481  LTWYQYHSLDVIGFLLAIVLGVAFIAFKCCAYGCRKCFGKKGSVKKGHKSKAH  533
```

5. UGT1A9 alignment between

Query: NCBI Reference Sequence: NP_066307.1; UDP-glucuronosyltransferase 1-9 precursor [Homo sapiens]

Sbjct: NCBI Reference Sequence: XP_015337084.1; PREDICTED: UDP-glucuronosyltransferase 1-9 isoform X3 [Marmota marmota marmota]

```
Identities      Positives      Gaps
429/521(82%)    463/521(88%)   0/521(0%)

Query  10   LPLCVCLLLTCGFAEAGKLLVVPMDGSHWFTMRSVVEKLILRGHEVVVVMPEVSWQLGRS  69
            LPLCVCLLL    A+AGKLLVVPMDGSHWFTMRSVVEKL+ RG+EVV VMPEVSWQLG+S
Sbjct  10   LPLCVCLLLASSSAQAGKLLVVPMDGSHWFTMRSVVEKLVHRGNEVVAVMPEVSWQLGQS  69

Query  70   LNCTVKTYSTSYTLEDLDREFKAFAHAQWKAQVRSIYSLLMGSYNDIFDLFFSNCRSLFK  129
            LN TVKTYSTSYTLEDLDR F  F   QWK   +S+YS  MGS    FD+ FS CRSLF
Sbjct  70   LNFTVKTYSTSYTLEDLDRGFNFFVDTQWKTPEQSMYSAAMGSSKAFFDITFSRCRSLFN  129

Query  130  DKKLVEYLKESSFDAVFLDPFDNCGLIVAKYFSLPSVVFARGILCHYLEEGAQCPAPLSY  189
            DKKLVEYLKE+SFDAVFLDPFD CGL+VAKYFSLPSVVFAR + C++LE+GAQCP+PLSY
Sbjct  130  DKKLVEYLKETSFDAVFLDPFDVCGLVVAKYFSLPSVVFARVVFCNFLEDGAQCPSPLSY  189

Query  190  VPRILLGFSDAMTFKERVRNHIMHLEEHLLCHRFFKNALEIASEILQTPVTEYDLYSHTS  249
            VPR+ L  SDA++F ER+RNH+ +LEE+L C  FF+ ALE+ASEIL+TPVT  DL+S  S
Sbjct  190  VPRVFLMSSDALSFMERIRNHLNYLEEYLFCPYFFQTALEVASEILRTPVTIGDLFSQIS  249

Query  250  IWLLRTDFVLDYPKPVMPNMIFIGGINCHQGKPLPMEFEAYINASGEHGIVVFSLGSMVS  309
            IWLLRTDFVL+YP+PVMPNMIF+GGINCHQ KPLP EFEAY+NASGEHGIVVFSLGSMVS
Sbjct  250  IWLLRTDFVLEYPRPVMPNMIFVGGINCHQRKPLPKEFEAYVNASGEHGIVVFSLGSMVS  309

Query  310  EIPEKKAMAIADALGKIPQTVLWRYTGTRPSNLANNTILVKWLPQNDLLGHPMTRAFITH  369
            EIPEKKAM IADALG+IPQTVLWRYTGT PSNLA NT LVKWLPQNDLLGHP TRAFITH
Sbjct  310  EIPEKKAMEIADALGRIPQTVLWRYTGTPPSNLAKNTKLVKWLPQNDLLGHPKTRAFITH  369

Query  370  AGSHGVYESICNGVPMVMMPLFGDQMDNAKRMETKGAGVTLNVLEMTSEDLENALKAVIN  429
            AGSHGVYE ICNGVPMVMMPLFGDQMDNAKRMET+GAGVTLNVLEMTS+DL NALKAVIN
Sbjct  370  AGSHGVYEGICNGVPMVMMPLFGDQMDNAKRMETRGAGVTLNVLEMTSDDLANALKAVIN  429

Query  430  DKSYKENIMRLSSLHKDRPVEPLDLAVFWVEFVMRHKGAPHLRPAAHDLTWYQYHSLDVI  489
            DKSYKENIMRLS LHKDRP+EPLDLAVFWVEFVMRHKGAPHLRPAAHDLTWYQYHSLDVI
Sbjct  430  DKSYKENIMRLSRLHKDRPIEPLDLAVFWVEFVMRHKGAPHLRPAAHDLTWYQYHSLDVI  489

Query  490  GFLLAVVLTVAFITFKCCAYGYRKCLGKKGRVKKAHKSKTH  530
            GFLLA+VL VAFI FKCCAYG RKC GKKG VKK HKSK H
Sbjct  490  GFLLAIVLGVAFIAFKCCAYGCRKCFGKKGSVKKGHKSKAH  530
```

6. UGT2B4 alignment between

Query: NCBI Reference Sequence: NP_066962.2; UDP-glucuronosyltransferase 2B4 isoform 1 precursor [Homo sapiens]

Sbjct: NCBI Reference Sequence: XP_015337650.1 PREDICTED: UDP-glucuronosyltransferase 2B4-like [Marmota marmota marmota]

```
Identities      Positives      Gaps
54/115(47%)     60/115(52%)    44/115(38%)
Query  250  MAKADIWLIRNYWDFQFPHPLLPNVEFVGGLHCKPAKPLPKEMEEFVQSSGENGVVVFSL  309
            MAKADIWLI  YWD +F H  LPNV+ VGGLHC+PAK LP
Sbjct  1    MAKADIWLICTYWDLEFSHSTLPNVDIVGGLHCRPAKSLP--------------------  40

Query  310  GSMVSNTSEERANVIASALAKIPQKVLWRFDGNKPDTLGLNTRLYKWIPQNDLLG  364
                                    K+LWRFDG KPDTLGLN +LYKWIP+ DLLG
Sbjct  41   ------------------------KILWRFDGKKPDTLGLNIQLYKWIPKGDLLG  71
```

7. UGT2B4 alignment (alternative)

Query: NCBI Reference Sequence: NP_066962.2; UDP-glucuronosyltransferase 2B4 isoform 1 precursor [Homo sapiens]

Sbjct: NCBI Reference Sequence: XP_015360783.1; PREDICTED: UDP-glucuronosyltransferase 2B15-like [Marmota marmota marmota]

```
Identities        Positives         Gaps
388/530(73%)      455/530(85%)      2/530(0%)

Query  1    MSMKWTSALLLIQLSCYFSSGSCGKVLVWPTEFSHWMNIKTILDELVQRGHEVTVLASSA  60
            MS+K  S LLLIQL CYFSSGSCGKVLVWPTE+SHW+NIK ILDEL+QRGHEVTVL S+A
Sbjct  1    MSVKRNSVLLLIQLMCYFSSGSCGKVLVWPTEYSHWINIKAILDELLQRGHEVTVLTSTA  60

Query  61   SISFDPNSPSTLKFEVYPVSLTKTEFEDIIKQLVKRWA-ELPKDTFWSYFSQVQEIMWTF  119
            SI  +PN+ S + FEVYP   +K   E+   + +  W  + PKD FW ++S VQ++   +
Sbjct  61   SILINPNTTSAINFEVYPAPSSKQHLEERFSKWIHEWIYDTPKDDFWEFYSLVQKVFKDY  120

Query  120  NDILRKFCKDIVSNKKLMKKLQESRFDVVLADAVFPFGELLAELLKIPFVYSLRFSPGYA  179
            +D + + C+++V NKKLM KL ES+FDVVLADAV P GELLAELLKIPFVY+LRF+ GY
Sbjct  121  SDTIEQLCRNVVLNKKLMMKLHESKFDVVLADAVGPCGELLAELLKIPFVYTLRFTFGYT  180

Query  180  IEKHSGGLLFPPSYVPVVMSELSDQMTFIERVKNMIYVLYFEFWFQIFDMKKWDQFYSEV  239
             EK+SGGL  PPSYVP+VMSELSDQMTF+ERVKN +Y+LYF+FWFQIFD+K+W+QFYSEV
Sbjct  181  YEKYSGGLTVPPSYVPIVMSELSDQMTFMERVKNTVYMLYFDFWFQIFDVKRWNQFYSEV  240

Query  240  LGRPTTLSETMAKADIWLIRNYWDFQFPHPLLPNVEFVGGLHCKPAKPLPKEMEEFVQSS  299
            LGRPTT+ ETM KAD WLIR YWD +FP PLLPN +FVGGLHCKPAKPLPKEMEEFVQSS
Sbjct  241  LGRPTTIYETMGKADFWLIRTYWDLEFPRPLLPNFDFVGGLHCKPAKPLPKEMEEFVQSS  300

Query  300  GENGVVVFSLGSMVSNTSEERANVIASALAKIPQKVLWRFDGNKPDTLGLNTRLYKWIPQ  359
            GENG+VVFSLG+MVSN  EE+AN+IA ALA+IPQKV+WRF+G KPD LG NT++Y+WIPQ
Sbjct  301  GENGIVVFSLGTMVSNMPEEKANMIAFALAQIPQKVIWRFNGKKPDKLGPNTQIYEWIPQ  360

Query  360  NDLLGHPKTRAFITHGGANGIYEAIYHGIPMVGVPLFADQPDNIAHMKAKGAAVSLDFHT  419
            NDLLGHPKT+AFITHGG NG+YEAIYHG+PMVG+PLFADQPDNIAH+KAKGAA+ LD+
Sbjct  361  NDLLGHPKTKAFITHGGTNGVYEAIYHGVPMVGIPLFADQPDNIAHVKAKGAAIRLDYRI  420

Query  420  MSSTDLLNALKTVINDPLYKENAMKLSRIHHDQPVKPLDRAVFWIEFVMRHKGAKHLRVA  479
            ++S DLL AL+ VINDP YKENAM+LSRI HDQPVKPLDRAVFWIEFVMRHKGAKHL+VA
Sbjct  421  LTSADLLKALRMVINDPSYKENAMELSRIQHDQPVKPLDRAVFWIEFVMRHKGAKHLQVA  480

Query  480  AHDLTWFQYHSLDVTGFLLACVATVIFIITK-CLFCVWKFVRTGKKGKRD  528
            AHDL+WFQY+SLDV GFLLACVATV+FII+K CLFC   F +TGKK KR+
Sbjct  481  AHDLSWFQYYSLDVIGFLLACVATVMFIISKCCLFCFQMFFKTGKKEKRE  530
```

8. UGT2B15 alignment

```
Query: NCBI Reference Sequence: NP_001067.2; UDP-glucuronosyltransferase 2B15
precursor [Homo sapiens]

Sbjct: NCBI Reference Sequence: XP_015360783.1; PREDICTED: UDP-
glucuronosyltransferase 2B15-like [Marmota marmota marmota]


Identities        Positives        Gaps
392/530(74%)      459/530(86%)     0/530(0%)

Query  1    MSLKWTSVFLLIQLSCYFSSGSCGKVLVWPTEYSHWINMKTILEELVQRGHEVTVLTSSA  60
            MS+K  SV LLIQL CYFSSGSCGKVLVWPTEYSHWIN+K IL+EL+QRGHEVTVLTS+A
Sbjct  1    MSVKRNSVLLLIQLMCYFSSGSCGKVLVWPTEYSHWINIKAILDELLQRGHEVTVLTSTA  60

Query  61   STLVNASKSSAIKLEVYPTSLTKNYLEDSLLKILDRWIYGVSKNTFWSYFSQLQELCWEY  120
            S L+N + +SAI  EVYP   +K +LE+   K +  WIY   K+ FW ++S +Q++  +Y
Sbjct  61   SILINPNTTSAINFEVYPAPSSKQHLEERFSKWIHEWIYDTPKDDFWEFYSLVQKVFKDY  120

Query  121  YDYSNKLCKDAVLNKKLMMKLQESKFDVILADALNPCGELLAELFNIPFLYSLRFSVGYT  180
             D   +LC++ VLNKKLMMKL ESKFDV+LADA+ PCGELLAEL  IPF+Y+LRF+ GYT
Sbjct  121  SDTIEQLCRNVVLNKKLMMKLHESKFDVVLADAVGPCGELLAELLKIPFVYTLRFTFGYT  180

Query  181  FEKNGGGFLFPPSYVPVVMSELSDQMIFMERIKNMIHMLYFDFWFQIYDLKKWDQFYSEV  240
            +EK  GG   PPSYVP+VMSELSDQM FMER+KN ++MLYFDFWFQI+D+K+W+QFYSEV
Sbjct  181  YEKYSGGLTVPPSYVPIVMSELSDQMTFMERVKNTVYMLYFDFWFQIFDVKRWNQFYSEV  240

Query  241  LGRPTTLFETMGKAEMWLIRTYWDFEFPRPFLPNVDFVGGLHCKPAKPLPKEMEEFVQSS  300
            LGRPTT++ETMGKA+ WLIRTYWD EFPRP LPN DFVGGLHCKPAKPLPKEMEEFVQSS
Sbjct  241  LGRPTTIYETMGKADFWLIRTYWDLEFPRPLLPNFDFVGGLHCKPAKPLPKEMEEFVQSS  300

Query  301  GENGIVVFSLGSMISNMSEESANMIASALAQIPQKVLWRFDGKKPNTLGSNTRLYKWLPQ  360
            GENGIVVFSLG+M+SNM EE ANMIA ALAQIPQKV+WRF+GKKP+ LG NT++Y+W+PQ
Sbjct  301  GENGIVVFSLGTMVSNMPEEKANMIAFALAQIPQKVIWRFNGKKPDKLGPNTQIYEWIPQ  360

Query  361  NDLLGHPKTKAFITHGGTNGIYEAIYHGIPMVGIPLFADQHDNIAHMKAKGAALSVDIRT  420
            NDLLGHPKTKAFITHGGTNG+YEAIYHG+PMVGIPLFADQ DNIAH+KAKGAA+ +D R
Sbjct  361  NDLLGHPKTKAFITHGGTNGVYEAIYHGVPMVGIPLFADQPDNIAHVKAKGAAIRLDYRI  420

Query  421  MSSRDLLNALKSVINDPVYKENVMKLSRIHHDQPMKPLDRAVFWIEFVMRHKGAKHLRVA  480
            ++S DLL AL+ VINDP YKEN M+LSRI HDQP+KPLDRAVFWIEFVMRHKGAKHL+VA
Sbjct  421  LTSADLLKALRMVINDPSYKENAMELSRIQHDQPVKPLDRAVFWIEFVMRHKGAKHLQVA  480

Query  481  AHNLTWIQYHSLDVIAFLLACVATVIFIITKFCLFCFRKLAKKGKKKKRD  530
            AH+L+W QY+SLDVI FLLACVATV+FII+K CLFCF+   K GKK+KR+
Sbjct  481  AHDLSWFQYYSLDVIGFLLACVATVMFIISKCCLFCFQMFFKTGKKEKRE  530
```

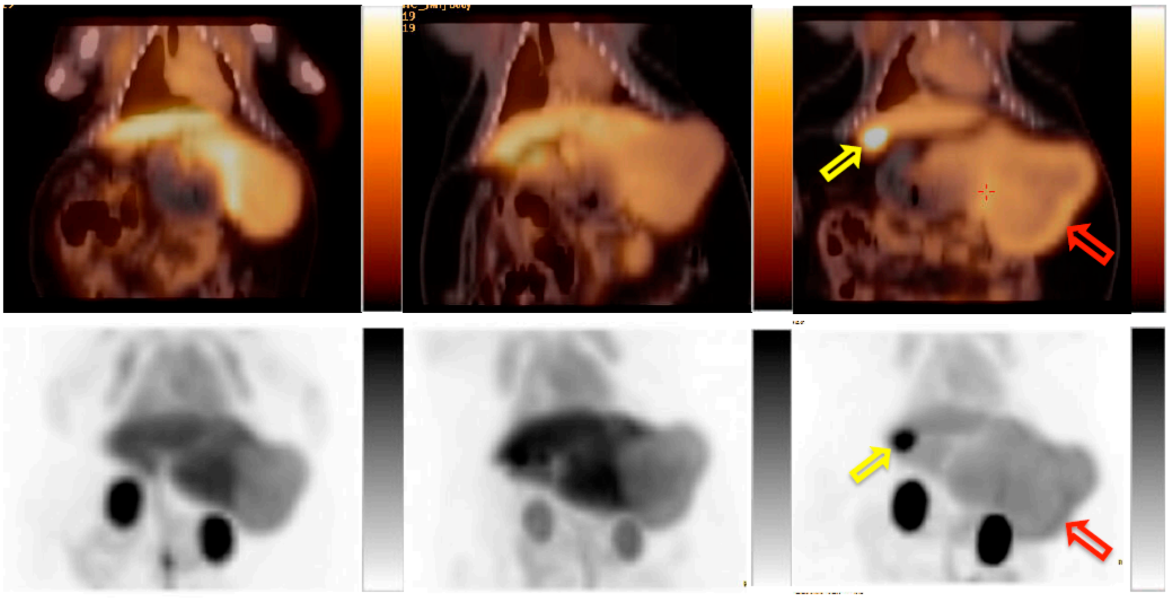

Animal C: In this animal, liver background uptake is much higher than tumor uptake. Only after cold CFA application, the uptake around the tumor rim (red arrow) can be perceived. Gallbladder (GB, yellow arrow) is clearly displayed from the PET/CT overlay and MIP (yellow arrow) with cold CFA application, which can be weakly identified from the middle panel with probenecid application.

Figure S2. Three-way comparison of [18F]CFA uptake in tumor (red arrow) and liver. From the left: baseline, post UGT inhibitor probenecid application, and after cold CFA application. In each column, the upper panel is PET/CT overlay while the lower panel is the maximal intensity projection (MIP).

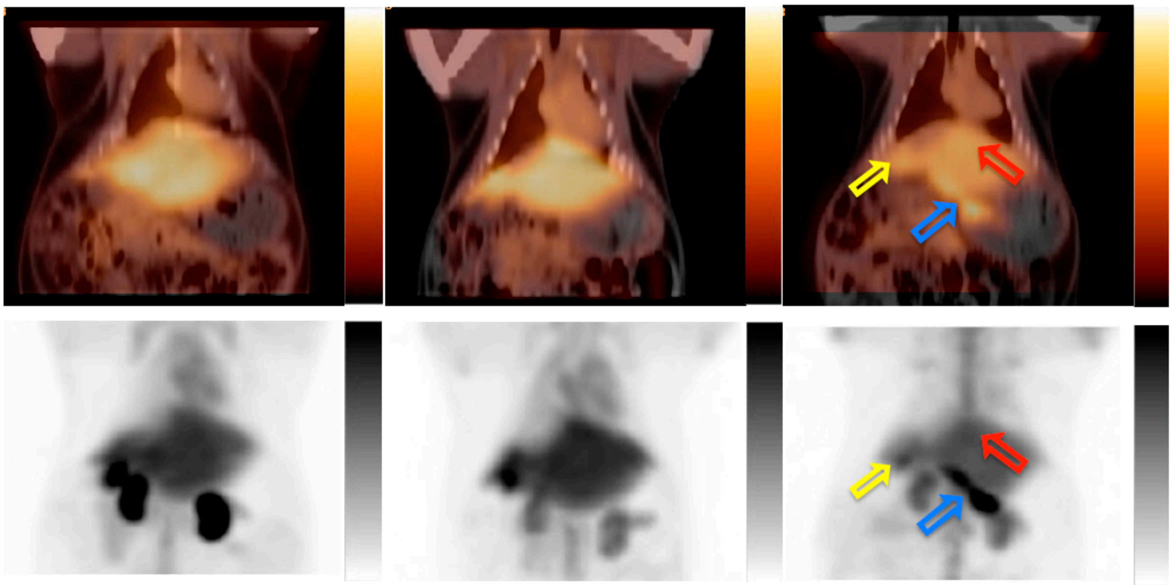

Animal D: Right column: Gallbladder (GB, yellow arrow) can be discerned from the image after cold CFA application. Cold CFA application also caused strong intestinal accumulation (blue arrow).

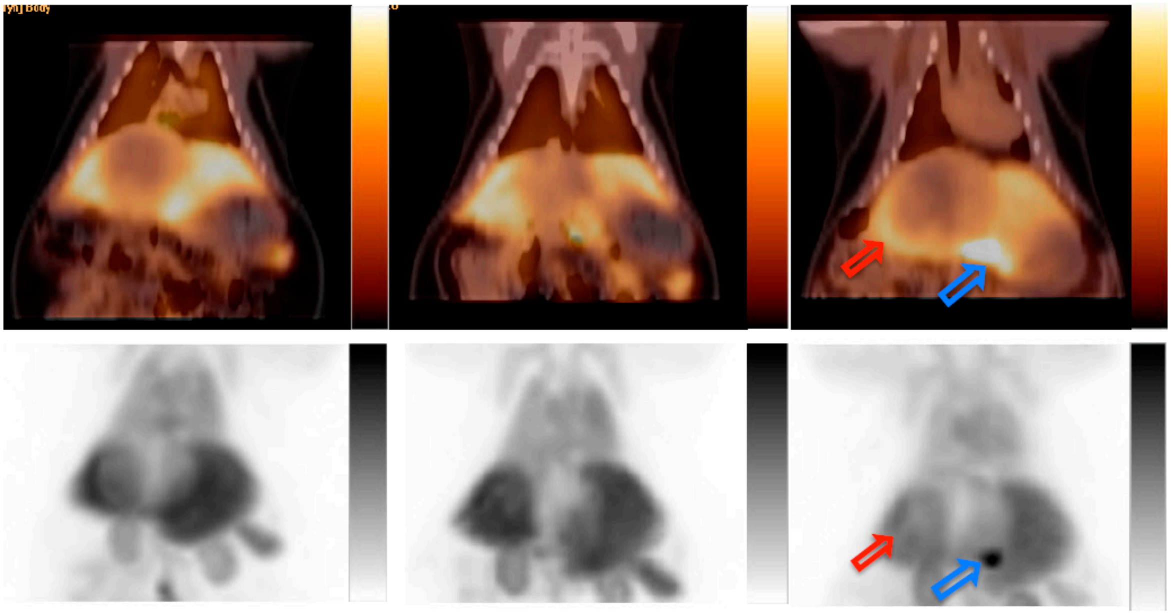

Animal E: The tumor's uptake in the proliferative rim (red arrow) with no GB but intestinal accumulation.

Figure S3. Three-way comparison of [^{18}F]CFA uptake in tumor (red arrow) and liver. From the left: baseline, post PNP inhibitor forodesine application, and after cold CFA application. In each column, the upper panel is PET/CT overlay while the lower panel is the maximal intensity projection (MIP).
